# From Choose and Hope to Evidence and Impact: Rethinking School-Based Behavioral Health Decisions

**DOI:** 10.3390/bs16081319

**Published:** 2026-08-03

**Authors:** Joseph Latimer, Nathaniel von der Embse, Katlyn Reynolds, Hannah Brady, Melissa Brown, Krysta Salvato-Hayes, Jennifer Wolgemuth, Stephen Kilgus

**Affiliations:** 1 School Mental Health Collaborative, College of Education, University of South Florida, Tampa, FL 33620, USA; 2 Department of Educational and Psychological Studies, College of Education, University of South Florida, Tampa, FL 33620, USA; natev@usf.edu (N.v.d.E.); katlynr1@usf.edu (K.R.); hannahbrady@usf.edu (H.B.); melissabrown@usf.edu (M.B.); ksalvato@usf.edu (K.S.-H.); jrwolgemuth@usf.edu (J.W.); 3 Department of Educational Psychology, School of Education, University of Wisconsin–Madison, Madison, WI 53706, USA; skilgus@wisc.edu

**Keywords:** school-based behavioral health, decision-making, implementation

## Abstract

A challenge in school-based behavioral health is how evidence-based practices are selected and implemented. Decision-making within schools often prioritizes feasibility instead of research evidence. The purpose of this study was to examine the experiences of school decision-makers in selecting and implementing evidence-based behavioral health programs. The study employed an interpretivist methodology, conducting interviews with 18 school decision-makers across K-12 school and district settings in Florida (n = 8), Kansas (n = 7), and Pennsylvania (n = 3). The study also included observations of 14 school meetings across Florida (n = 2), and Kansas (n = 12). The participants in Florida and Kansas were a part of the interviews and observations. The data collected were analyzed using an iterative diagrammatical analysis, which created the STAR (Select, Trial, Assess, Revise) Model of Behavioral Health Implementation in Schools. This model is a non-linear framework that captures the phases of behavioral health support implementation in schools. The model components are discussed, including examples from school decision-makers. The findings of this study have implications such as (a) a novel methodology for examining school-based decision-making, (b) a new model to conceptualize and enact evidence-based practices, and (c) future studies to amplify the study’s findings.

## 1. Introduction

Approximately one in five children in the United States experience a mental, emotional, or behavioral health need (Centers for Disease Control and Prevention, 2025). However, this is likely an underestimation, as it underrepresents subclinical and undiagnosed cases (Bitsko et al., 2022). Despite the availability of evidence-based interventions, children and families face many barriers to accessing behavioral healthcare (von der Embse et al., 2021). These barriers include costs, limited provider availability, poor quality, social stigma, perceptions of embarrassment or fear of judgment, and a lack of awareness (Aguirre Velasco et al., 2020; Gunlicks-Stoessel et al., 2025). As a result, many youth do not receive timely or adequate support. Given these widespread logistical and systemic barriers, schools are uniquely positioned to serve as a primary setting for delivering mental and behavioral health services (R. D. Taylor et al., 2017). These services may include academic, social, and emotional interventions, which may address a wide range of internalizing and/or externalizing needs. They also allow for early identification and intervention in a natural setting where academic, behavioral, and social-emotional needs overlap (Splett et al., 2017).

To address the behavioral and mental health needs of students, schools increasingly rely on comprehensive, system-level frameworks such as Multi-Tiered Systems of Support (MTSS) and prevention science models for reducing behavior (Sugai & Horner, 2009). These approaches emphasize a range of supports across universal (Tier 1), targeted (Tier 2), and intensive (Tier 3) levels, with a focus on early identification, data-based decision-making, and proactive intervention (Sugai & Horner, 2009). Within the context of mental and behavioral health supports, MTSS is used as the framework for organizing prevention and intervention services along a continuum of intensity matched to the needs of students. Prevention science further supports this model by prioritizing the reduction in risk factors and the strengthening of protective factors before more severe behaviors occur. Rather than relying solely on individual interventions, these frameworks require coordinated, school-wide systems that align resources, personnel, and practices to effectively support student needs. Within these systems, behavioral frameworks such as Positive Behavioral Interventions and Supports (PBIS) are often used to organize and deliver supports (Center on Positive Behavioral Interventions and Supports, 2025). When implemented with fidelity, these frameworks have been associated with improvements in student behavioral, academic, and social-emotional outcomes (Bradshaw et al., 2012). However, effectiveness is dependent on the extent to which they are implemented in ways that align with the local context and available resources (Bradshaw et al., 2010).

A key challenge in selecting evidence-based interventions is the variation in the process used to select and the evidence used (or not) to support said choices (Neal et al., 2019). Although schools are expected to use evidence-based interventions (U.S. Department of Education, 2025), decisions are often influenced more by feasibility, acceptability, and available resources than by research alone. For instance, Neal et al. (2019) found that school decision-makers frequently prioritize what is practical and fits within existing systems over the strength of the research when choosing interventions. However, evidence-based practices are not always used as intended (Fox et al., 2022). For instance, many factors also impact the adoption and implementation of evidence-based practices such as administration support, implementer support and effective training, sustained financial resources and alignment with the practice to everyday policies (Forman et al., 2009). Thus, the combination of both the practice itself and the local context in which implementation occurs is a key consideration when it comes to sustainable adoption.

### 1.1. Research Evidence Use Within Mental and Behavioral Health Services in Schools

The success of prevention science initiatives, including MTSS, rests on the implementation of evidence-based practices (Cook et al., 2015). School districts are required by federal legislation and policy (U.S. Department of Education, 2025) to employ research-based practices; however, there continue to be substantial barriers to identifying and implementing these practices (Lyon & Bruns, 2019). First, educators often engage in decision-making constrained by bounded rationality (Lunenburg, 2010). That is, decisions are made without complete data or consideration of all possible alternatives. For example, convenience and immediacy may be more important drivers of decision-making than optimized outcomes. The bounded rationality model suggests that school teams cannot identify all possible choices and subsequent outcomes of said choice and rather will “choose and hope” without a complete analysis of options. This process inevitably results in decisions that may not be fully aligned to local resources, personnel, student population, and/or ideal outcomes. Moreover, empirical evidence alone is likely to be insufficient to determine the “correct” course of action, as value disagreements (e.g., principal prioritizing cost, teacher prioritizing time) may complicate the problem-solving process. Indeed, research has demonstrated the influence of external variables (policy or legislative mandates; Allen et al., 2018) and competing personnel and resource constraints on the decision-making process.

Second, the prioritization and use of research evidence in decision-making is often influenced by individual beliefs and attitudes towards research (Neal et al., 2022). Although online clearinghouses (e.g., What Works Clearinghouse) provide summaries and indicators of evidence quality, there is limited information on how a specific tool or intervention may be adapted to a certain school population or a setting with differing levels of resources (Neugebauer et al., 2021). For instance, a school team may identify an evidence-based program, but the localized resources (e.g., time, staff, training) are not sufficient to support adoption and implementation. This results in successfully identifying an evidence-based program with limited ability for implementation. The implementation science movement has attempted to bridge this gap by developing and communicating actionable and defensible guidance on how to apply, generalize, and maintain based on existing evidence (Fixsen et al., 2024). However, without specific accounting for local constraints in a systematic manner (von der Embse et al., 2021), these efforts may be limited given the wide variability in resources, values, and personnel within and between schools. Thus, there exists guidance for whether and how an intervention may work, but limited evidence for where (Orr et al., 2019).

Finally, barriers to effective decision-making and eventual implementation are often not limited by access to knowledge (e.g., coaching, technical assistance) per se, but rather the coordination and application of said knowledge (Chorpita & Daleiden, 2018). For instance, schools often rely on job-embedded coaching to gain skills and knowledge to implement coordinated systems of care in schools (Castillo et al., 2024). Yet, this process requires consistent and intentional engagement with the knowledge gained in coordination with the local context to allow for long-lasting change within a school (Freeman et al., 2017). Previous research into these processes has largely utilized quantitative, survey-based, and network analytic approaches (e.g., Neal et al., 2019). Qualitative approaches focused specifically on the decision-making processes as shaped by contextual, environmental, and interpersonal influences may provide important insight to localize future decision-making frameworks (Elliott & Timulak, 2005). Insight into the acquisition, synthesis, and application of evidence as it relates to real-world decision-making under local constraints may result in a more nuanced and localized application of evidence-based practices within mental and behavioral health.

### 1.2. Implementation Frameworks in Schools

In efforts to implement systems of support within schools, school decision-makers are mandated to follow the treatment frameworks of MTSS and PBIS as noted earlier within the paper (Sugai & Horner, 2009; Center on Positive Behavioral Interventions and Supports, 2025; U.S. Department of Education, 2025). However, these frameworks provide a contextual approach to navigating school systems and treatment approaches. There is an underlying research realm (i.e., implementation science) on how practices within these initiatives are put into real-world change (Fixsen et al., 2024). Within the educational world, there are some commonly cited frameworks for implementation science such as the active implementation framework (Fixsen et al., 2024) and the EPIS model (Exploration, Preparation, Implementation and Sustainment; Aarons et al., 2011). Across these models, there are commonalities, such as a sequenced process for change (e.g., plan, prep, implement, review), the need for specific factors to promote implementation (i.e., organizational leaders, implementation drivers) and the realization that there are non-linear pathways for change.

The implementation science frameworks reviewed delineate the key practices needed for effective adoption efforts. To date, these frameworks have largely been developed outside resource-constrained conditions and are optimized for installation across the largest number of schools and conditions. What is less clear is how schools with limited resources, competing priorities, and varying policy mandates may decide to adopt (or not) portions of these frameworks. As such, this study is theoretically anchored in a bounded rationality model that explicitly recognizes that many school-based decisions are unlikely to be optimized for a particular outcome as it is not possible to critically evaluate all options. Thus, the present study does not seek to relabel existing implementation steps but rather asks how school decision-makers go through various stages of negotiating the research evidence-to-practice gap within resource-constrained contexts.

### 1.3. Purpose of the Study

Given the importance of addressing youth mental health challenges in a systematic, research-based, and effective manner, it is critical to examine the experiences of school decision-makers’ decision-making processes when selecting and implementing mental and behavioral health supports within school-based settings. Specifically, outlining real-world experiences of how school decision-makers can navigate addressing youth challenges within bounded rationality may create insights for future research and guidance on translating evidence-based programs into school systems of support. The purpose of this study was to conduct a qualitative inquiry to examine how school decision-makers conceptualize and enact decision-making processes when selecting and implementing mental and behavioral health services within school-based MTSS and develop a nuanced depiction of decision-making grounded in decision-makers’ experiences, values, and contexts.

## 2. Materials and Methods

The study was situated within a broader research-practice collaboration focused on aligning research evidence, contextual constraints, and resource allocation in behavioral service delivery. The aim was to model participants’ reasoning processes, contextual influences, and values that shape implementation decisions in natural contexts. A qualitative approach was adopted consisting of semi-structured interviews with MTSS decision-makers and structured observations of MTSS meetings in alignment with our interpretivist qualitative methodology which sought subjective and naturalistic accounts (Elliott & Timulak, 2005). For instance, the research team viewed decision-making as socially constructed and context-embedded. The data collected within the interviews and observations overlapped. For instance, the observations occurred within one school district in Florida and one in Kansas. Many of the same individuals who were a part of the Florida and Kansas observations were also interviewed. The only participants who did not have overlap between the interviews and observations were those from Pennsylvania. Those individuals were only interviewed. Finally, it is important to note that there was an imbalance between interviews and observations. The study team relied more on the information gleaned from interviews as opposed to the observations, which created an imbalance between data sources.

### 2.1. Interview Participants

Participants included 18 school decision-makers in semi-structured interviews. The participants were purposefully selected to maximize variation (Patton, 2015) in geography, role, and experiences with MTSS. Inclusion criteria required participants to hold influence over intervention programming or student-support decisions within the educational environment.

Roles of participants interviewed included administrators (n = 6), student-service directors (n = 2), behavioral coaches (n = 5), school psychologists (n = 1), school counselors (n = 3) and a literacy coach (n = 1). Participants ranged in their previous years of experience working within MTSS frameworks or mental and behavioral health from 8 years to over 30 years, with many falling in the 10–15 years of experience. Participants were based in either elementary school settings, middle school settings, K-8 schools or at the district level. Overall, the gender breakdown was one male and 17 females.

Participants were employed by schools and districts in Kansas (n = 7), Pennsylvania (n = 3), and Florida (n = 8); three states differing in policy mandates, initiatives, and MTSS maturity. The participants in Kansas were employed by a suburban district serving a population of predominantly students who identify as white (over 60%). The participants from Florida were employed by a large urban district serving a more diverse population of students (i.e., approximately 40% Hispanic, 30% White, 20% Black, 5% Asian, and 5% multiracial). The participants from Pennsylvania were employed by a small rural district serving a population of predominantly students who identify as white (over 60%).

### 2.2. Observations

The research team conducted a total of 14 observations of MTSS meetings in seven schools. Similar to the interview participants, these schools were selected to maximize variation in geography, school type, and experience with MTSS. Six were public and one charter elementary school across Kansas and Florida. Although interviews were conducted with participants in Pennsylvania, observations were not completed due to limited interest. Each school team was observed during their pre-determined MTSS meeting time twice during the 2024–2025 and 2025–2026 school years. The team chose which MTSS meeting to observe in consultation with the school MTSS team and based on the criteria that they were decision-making meetings and involved interdisciplinary teams that addressed student data, intervention effectiveness, and resource allocation. To maintain observer consistency, each observer was trained prior to any completed observation, and a quality check of the observation protocol was completed after each observation.

### 2.3. Procedures

#### 2.3.1. Sampling

A purposeful sampling technique was utilized to recruit school decision-makers and school teams to engage in interviews and observations. The study is a part of a national research and technical assistance center for school-based mental health that encompasses multiple pre-existing research-practice partnerships. Thus, the study team reached out to the previously established partners to recruit for the study, which resulted in a 100% acceptance rate. The study team also purposefully recruited participants from geographically different areas and settings (i.e., urban, rural, suburban) to model variation in the implementation of mental and behavioral health supports in schools.

#### 2.3.2. Interview Procedures

Following Institutional Review Board (IRB) approval, participants were recruited through professional networks and district partnerships. Each received a study overview, emphasizing voluntary participation, confidentiality, and the opportunity to reflect on their decision-making practices. Written informed consent was obtained prior to all data collection. Semi-structured interviews served as the primary data source. Each interview lasted approximately 60–90 min and was conducted virtually by research team members with expertise in MTSS, which enabled interviewers to quickly establish collegiality and rapport, important to generating quality interview data (Roulston, 2010). The interview protocol (see Supplemental Materials) elicited narratives about identifying behavioral-health needs, evaluating evidence, weighing contextual constraints, and making service-implementation decisions. All interviews were audio-recorded, transcribed verbatim, edited for accuracy, and de-identified. This was done using the Microsoft Teams platform and Otter.ai.

#### 2.3.3. Observation Procedures

To complement the interview data with descriptions of real-time decision-making, the team conducted 14 in-person observations of MTSS meetings. Consent of all those within each MTSS team was obtained prior to the observation. Similar to the interviews, research members with expertise in MTSS conducted each observation and were trained prior to the observation. The observation protocol consisted of two sections (see Supplemental Materials; File S2). The first section was a Tier I/II modified from a previously developed checklist from a national MTSS research and technical assistance center. The purpose of this section was to gather a checklist of components typically expected to be present within a Tier I/II MTSS meeting. For instance, observers had to identify the personnel within the meeting and then evaluate the extent to which certain components of problem-solving were present or absent during the observation (i.e., data discussed, hypotheses generated, action plan determined). The observer would mark if that person or component was present and provide evidence via field notes. This data provided the study team with a sense of each team’s ability to align their meeting processes with the tenets of the MTSS framework. The second section was intended to capture more in-depth notes on the flow of the meeting, the kinds of mental health interventions and assessments discussed, and descriptions of research leveraged in decision-making. For instance, observers detailed the purpose and actions within the meeting, the decisions made from the meeting, and the integration of research evidence in aspects of the meeting. The observer was to first write down initial reactions to be later cleaned and expanded upon within the same day of the observation. Overall, observation sessions lasted between 30 and 60 min, with limited variability in meeting length. Post-observation memos summarized participants’ roles, interactional patterns, and emergent themes, providing additional context for the diagrammatic analysis.

#### 2.3.4. Pedagogical Approach and Positionality

This study was informed by an interpretivist epistemology grounded in the understanding that meaning is contextually and socially situated (Elliott & Timulak, 2005). The research team viewed decision-making not as an isolated cognitive process but as one shaped by institutional, professional, and interpersonal influences. Inquiry, therefore, centered on how participants constructed meaning within the systems and relationships that define professional environments.

Consistent with interpretivist qualitative inquiry, the interdisciplinary research team brought to the study diverse professional backgrounds, experiences, and expertise that informed both individual and collective interpretations. The team included scholars and practitioners in school psychology, special education, and qualitative research, each with experience in implementing and researching MTSS and mental and behavioral health supports in schools. Additionally, many authors work within a national research and technical assistance center focused on improving MTSS for mental and behavioral health, regularly collaborating with schools to support implementation and systems improvement. These professional experiences informed how the team understood participants’ descriptions of decision-making and implementation.

First, the study team has an idealistic and optimistic viewpoint regarding the use of an MTSS framework for school-based mental and behavioral health. Thus, there was an underlying assumption that MTSS represents an effective and efficient method for supporting youth. This assumption may have influenced how the team approaches data collection and interpretation and may have contributed to participants perceiving the researchers as supportive of MTSS initiatives. Similarly, the team’s prior experiences may have resulted in a more favorable interpretive viewpoint during observations.

Throughout data analysis, the research team encountered participant perspectives that differed from the team’s initial assumptions. While several team members entered the study viewing MTSS as an effective framework for organizing school-based mental health supports, participants often described the practical complexities of implementation, including competing priorities, limited resources, shifting policies, and contextual constraints. These accounts prompted ongoing discussion among the research team regarding how prior professional experiences might shape interpretation and reinforced the importance of grounding analytic decisions in participants’ descriptions rather than researchers’ expectations.

The team remained reflexive throughout data collection and analysis by documenting analytic memos and engaging in regular discussions regarding subjectivities, positionalities, and emerging interpretations (Roulston & Shelton, 2015). Team discussions served as structured opportunities to interrogate assumptions, consider alternative explanations, and distinguish participants’ accounts from researchers’ prior professional experiences. Ultimately, the team’s reflexive stance positioned the researchers as active learners engaged in understanding, rather than judging, participants’ decision-making processes within their unique organizational contexts.

### 2.4. Data Analysis

Given the aim was to build a decision-making model grounded in interview and observation data, the five-person qualitative analysis team adopted a visual, diagrammatic approach called Iterative Diagrammatical Analysis (IDA; see Wolgemuth et al., n.d., for a full description of the IDA process). Iterative Diagrammatical Analysis is a non-coding approach to qualitative data analysis (Wolgemuth et al., 2024) in which diagramming and diagrammatical reasoning (Peirce, 1934) serve as the primary analysis strategy. This approach was chosen over conventional qualitative approaches such as coding and thematic analysis (Miles et al., 2014; Saldaña, 2021) given diagramming’s alignment with the project’s overall aim to produce a mental-health decision-making model based on the data; the usefulness of drawing, doodling, and diagramming for reasoning, understanding, and interpreting text (e.g., Kenning, 2021); and diagramming’s holism in its depiction of relational, temporal, and contextual dynamics of social processes. The IDA unfolded through three iterative phases over the course of 18 weeks (one interview per week): (a) exploratory diagramming, (b) comparative diagramming, and (c) finalizing and validating. In the exploratory diagramming phase, each of the five analysis team members immersed themselves in the week’s interview using data engagement practices common to qualitative research including reading, memo writing, and annotation (e.g., Mihas, 2025; Shelton & Coogler, 2025), placing diagramming at the center of analytic activity. Each analysis team member’s early diagrams were informal drawings (Jellema et al., 2022) or doodles (Quinn, 2021) which functioned as visual memos of emerging processes and relationships. The team drew on Musgrove and Musgrove’s (2015) handmade thinking text that details 21 diagram types (e.g., metaphor, tree, web, process, and layer) and Verdinelli and Scagnoli’s (2013) data display typologies (e.g., matrix, network, flow chart) as inspiration for their exploratory diagrams. For example, one team member combined a metaphor and process diagram to explore the decision-making process of a participant who used the metaphor of an umbrella to describe her school’s trusting relationship with district leaders (see Figure S2). Jellema et al. (2022) argue that analytic drawing is “related to the senses directly more than words” and thus through diagramming each research team member accessed a “deeper level of cognition” (p. 1399). Just as coding extends researchers’ analytic engagement with data (Saldaña, 2021), the team’s initial diagramming prolonged their attention to relationships, sequences, and decision points in the participants’ descriptions of decision-making. Thus, all five analysis team members produced five unique initial models which they shared and compared at two-hour bi-weekly consensus meetings whose aim was to produce a consensus model. In these meetings the analytic team took turns sharing their models, what they represented, and reflexively shared the assumptions made, reactions, and what remained unclear. The team drafted and finalized collective diagrams on butcher paper and whiteboards, photographed and audio-recorded descriptions of them, and recreated them in Miro, a digital collaborative whiteboard platform, to facilitate further refinement, version control, and sharing across the research team. The digital format enabled continued iterative revision while preserving earlier versions of models for comparison over time. The digital format enabled continued iterative revision while preserving earlier versions of models for comparison over time (see example Miro collective diagram in Figure S3).

Once the team had completed four to six interview collective diagrams, they moved on to collaborative diagramming, dedicating meetings to compare structural similarities and contextual variations across the collective diagrams to produce an interim omnibus diagram that reflected the interviews analyzed (see Figure S4). The team repeated this process for two more rounds, one of which solicited feedback from the broader study team, until all 18 interviews had been analyzed (diagrammed). In the second and third rounds, the team compared previous omnibus models to newly created ones, iteratively creating and refining what would be the final omnibus model. The research team then reviewed the final model in comparison to the observation data, asking how the observation data aligned with the model and making adjustments accordingly. The resulting product was a conceptual model of school-based behavioral-health decision-making that depicted individual, team, and systemic influences.

### 2.5. Data Trustworthiness

The study team followed the guidance of Lincoln and Guba (1985) to implement multiple procedures to promote the credibility, dependability, confirmability, and transferability of the data. First, to promote the credibility of the data analysis process, the study team followed a standard interview protocol (File S1). After data collection, each member of the study reviewed the same interview and internal member checking and consensus generation with weekly meetings. Additionally, the consensus developed by the team was documented along with each individual model produced within the IDA approach. Second, in an effort to promote the dependability of the data, all models, notes, participant codes, and group consensus decisions were documented throughout the analysis process. The study team has an article under review with a qualitative methodological focus that provides a detailed outline of the analysis process. Thus, additional studies could mirror the methods and have similar conclusions from the data. Third, the study team acknowledges the influence of their positionality (see Section 2.3.4), which promotes the confirmability of the conclusions made from the data collected in the study. Last, the study team provided ample information on the sample, setting, sampling rationale, and connection between the findings and the words of real-world school decision-makers. This information can allow for transferability of the conclusions of the study into the real-world settings of school decision-makers.

## 3. Results

Through this iterative analytic process, the team developed the STAR Model of Behavioral Health Implementation in Schools (Select, Trial, Assess, Revise; see Figure S1 in Supplementary Materials). The STAR Model represents a non-linear, context-dependent framework that reflects how school teams in the study made and refined decisions about implementing mental and behavioral health supports. Rather than following a fixed sequence, the model illustrates that school teams can enter the decision-making process at any phase of the model, including Select, Trial, Assess, or Revise, reflecting the dynamic, adaptive nature of decision-making within school systems. The STAR model consists of two foundational components as well as four stages. Within each component and stage there are a variety of variables that impact the experience of the school decision-maker. Below are explanations of the two foundational components, the four stages, and examples of participants’ perspectives that guided model development. In alignment with the interpretive nature of this research, we have connections to current literature woven into the presentation of findings as well as later within the discussion section. Please refer to Tables S1 and S2 for more information on components and themes of the STAR model.

There are two important considerations for the STAR model to note. First, the model components that are described below situate whether and how school teams engage in the four stages, and that the stages themselves are recursive with differing entry points, backward movement, and repeated cycling. For example, trust and relationships operate within the Select stage as programs can still fail if implementers do not believe in it (Participant 12). Below, we describe how these elements interact throughout the decision-making process rather than describing them as isolated events, and we detail the movement of one team across stages to depict the model in action. Second, participants noted that the decision process is non-linear, which is reflected in the STAR Model. Participants often noted that when implementing an intervention program schools may (a) select an intervention program and not complete a trial implementation, expecting it to be integrated immediately, (b) get an intervention program to be piloted, but slowly lose priority based on conflicting mandates, (c) or be in a constant stage of revising an intervention that is not favorable due to the amount of investment in the program.

Finally, it is also important to note that the data from the observations provided limited comparisons to the interview data. For instance, the observations provide a snapshot of real-life team functioning, which has some connections to the model outlined below. However, (a) not all observers included descriptive memos and instead relied on check boxes to report whether something was observed (e.g., data-based decision-making); (b) the observations were taken during the second half of the school year; and (c) meetings observed often focused on singular issues (i.e., behavior instances that recently happened) or ongoing implementation monitoring. Thus, the data gleaned from the observations were mostly aligned with the latter stages of the STAR model.

### 3.1. Foundational Components

Within the STAR model, there are two foundational components that precede decision-making and implementation of mental and behavioral health supports within schools. Both components drive the decision-making and implementation process.

#### 3.1.1. Foundational Component #1: Core Elements

First are core elements, which are necessary conditions that support the adoption and transition of an idea into practice. These include (a) empowerment of teacher/implementers, (b) alignment between the intervention and school goals, (c) strong relationships, and (d) a culture of trust.

##### Empowerment of Teachers/Implementers

Participants consistently emphasized the importance of teacher ownership in implementing interventions. This finding was consistent with current research suggesting that teacher ownership and shared leadership are critical in school-based implementation efforts (Sugai & Horner, 2009). One participant highlighted empowerment as, “teachers to make the decision and have the knowledge base to make decisions to support their students because they know them best” (Participant 10). The important distinction of this theme was the notion that mental and behavioral health was “one more thing”, which amplified having teachers and implementers empowered to implement. For instance, many decision-makers recognized that asking teachers to implement programs is often viewed as outside their instructional curriculum responsibilities. Thus, individuals required ownership of a program to integrate into their daily functioning to simply allow for a change to occur.

##### Alignment

Mirroring examples of implementation science frameworks, participants described the need for a clear connection between new interventions and existing school goals, structures, and systems. This alignment allowed practices to be integrated rather than viewed as add-ons. Some participants acknowledged challenges aligning classroom systems with school-wide frameworks such as PBIS, explaining that teachers may “have an independent system in their classroom. However, they try…to link it to our school wide system” (Participant 10). Others emphasized the complexity of coordinating across multiple service providers, particularly aligning with IEP goals. One participant explained, “we’re trying to align that with whatever the BSEL (Behavioral and Social Emotional Learning) goals are on the IEP” (Participant 12). These examples illustrated that successful implementation requires intentional coordination, shared frameworks, and clear communication across levels of support, which aligns with prior work on integrated school-wide and tiered systems of support (McDaniel et al., 2022). Additionally, participants noted that having alignment with school or district goals supported community engagement and approval of a new program. Specifically, participants noted that the implementation of programs related to mental and behavioral health has been seen as a political issue, and the more aligned with the school mission a program is, the more favorable it is viewed by the broader community.

##### Relationships

Participants also described relationships as central to successful implementation, emphasizing that strong connections supported collaboration, communication, and fidelity. Many participants highlighted reliance on “local experts”, such as PBIS coaches, school psychologists, and social workers, to support teachers. Relationships extended to families as well, with efforts to strengthen family–school partnerships through initiatives such as shared meals and communication strategies. Participants also noted the specific skills of PBIS coaches, school psychologists, and social workers were critical due to the subject knowledge of the components of mental and behavioral programs. Specifically, teachers needed to be coached by or have a strong collaboration with someone who had a mental or behavioral health background to allow for effective implementation.

##### Trust

Trust emerged as a foundation for collaboration and implementation, which has been noted as a pillar in school-based implementation efforts (Castillo et al., 2024; Wilhelm et al., 2021). Participants described trusting administrators, colleagues, and local experts to guide decision-making and provide support. Participants also highlighted the importance of trusting the evidence base behind the interventions, questioning credibility and motivation. As one participant stated, “…is it from someone in education, a PhD? Is it in an academic journal? What are, what’s the data behind it?” (Participant 5). One key component of the idea of “trust” was that it often was a personal decision at the implementer level. The team noticed that many participants noted that they either (a) trusted a specific person in the district or school and/or (b) trusted the source (i.e., legitimate website) or communication (i.e., strong marketing) of a program. Given the vast amount of information and complex research findings, they only had the capacity to give programs the “eye-ball” test.

#### 3.1.2. Foundational Component #2: Need for Change

The second foundational component is a need for change, defined as a clear rationale for adopting a new practice. This need was often driven by (a) alignment with mission and vision, (b) identified problems or student need, (c) policy mandates, and (d) emerging or promising practices.

##### Aligning with the Mission and Vision of the School/District

Participants frequently connected interventions to the broader mission, vision, and core values of the school or district. Aligning new practices with priorities such as student well-being and academic success helped justify adoption and build staff understanding. Participants emphasized researching strategies to ensure alignment with district goals noting they would “determine what kind of a plan would best fit us as a district and move forward from there” (Participant 5).

##### Noted Problem or Specific Need

Participants indicated that a need for change was often initiated by observable issues, such as behavioral challenges, attendance concerns, or gaps in support. These problems served as strong drivers for implementation. One participant described examining data to identify needs, noting that:

“If attendance is an issue, you’re going to end up having some concerns, whether it’s because of mental health or because of something else…”

(Participant 1)

##### Policy and Mandates

Participants described adopting practices in response to federal and state requirements. These mandates required schools to implement interventions aligned with policy expectations and shaped both selection and design. One participant described evaluating whether programs could “meet these requirements” saying, “can they (i.e., components of a selected program) meet these requirements…?” (Participant 4).

##### Innovative or Promising Practice

Another motivator for implementation was the discovery of an innovative or promising practice that captured decision-makers’ interest. Participants outlined that often districts move towards the “shiny” thing each year without regard for its impact on staff capacity.

“We’ve had so many initiatives… And those shiny new things are great, but oftentimes we just don’t have the … capacity…”

(Participant 9)

### 3.2. STAR Stages

With the above foundational components in place, the STAR model reflects four stages of school-based mental health decision-making: Select, Trial, Assess, Revise. It is important to note that the Select stage of the model also has separate sub-stages that outline the decision-making process of selecting and planning for a mental or behavioral health program. Below is an outline of each stage.

#### 3.2.1. Stage 1: Select

The first stage of the STAR model is Select, which focuses on the selection and planning of mental or behavioral programs to implement. Within this stage, there are three sub-stages: information gathering, filtering, and final selection.

##### Sub-Stage #1: Information Gathering

Within the information gathering sub-stage, school decision-makers gather information from a variety of localized and online resources. First, some participants mentioned consulting with colleagues at their schools to receive input on interventions. This finding aligns with prior literature that highlights the role of collaborative problem-solving and professional networks in school-based implementation decision-making (Fullan et al., 2015). A participant shared, “And then oftentimes, our special education teachers, our behavior coaches, our school psychs, social workers, they have a lot of great, valuable input onto some interventions” (Participant 9). Second, participants discussed consulting with external partners and professional networks to collect information on interventions. One participant shared, “I would probably collaborate with a behavior coach that I’m close with…I probably connect more with my colleagues who are outside of the district than in the district” (Participant 12). This network of informal consultation suggests that external professional relationships serve as important sources of expertise, helping decision-makers evaluate and adopt new practices.

Third, participants emphasized the importance of researching across multiple sources, including reviewing articles, journals, online resources and a general internet search to determine whether a program has been effective in similar contexts. One participant stated, “I’m probably gonna do a Google Scholar search on research that’s been done… then probably analyze …what’s most similar.” (Participant 12).

Finally, participants described learning about new interventions and networking with peers at conferences and professional development events, using these opportunities to engage with experts and stay informed about broader educational trends. One participant shared, “I go to conferences, I read, I do my journals, I do my due diligence…” (Participant 10).

The result of any combination of information gathering is “verified information”, which is when a school decision-maker believes that enough information is gathered and aligned to move toward the second sub-stage, filtering.

##### Sub-Stage #2: Filter

This sub-stage focuses on school decision-makers filtering the “verified information” with local variables to come to a final selection (third sub-stage). The notion of “verified information” is when a school decision-maker filters the concept of an intervention program through any of the following factors: the local expertise available to implement this program, school and community values, the likelihood of the school community valuing the program, policies and laws within their educational setting, relationships across district and school staff, the values of parents within their community, current procedures and the resources available (i.e., time, money, staff availability, professional development necessary). Many of these findings mirror the facilitating factors that are commonly associated with the successful implementation of school-based interventions (Takens et al., 2024; Wilhelm et al., 2021). It is important to note that the “verification process” was often driven by subjective confidence, as opposed to evidence from research. For instance, if the program did not seem to be cumbersome or controversial to the local context, it was selected. Evidence of a program was assumed if it was commonly known by the decision-maker.

Regarding the local experience resources available, participants expressed that they evaluate their own experience and expertise, as well as that of staff responsible for implementation, when considering a potential program. For instance, one participant noted that staff rely on previous knowledge: “A lot of them [teachers] use… knowledge of what they have…I have this program…I’m just going to run the program’” (Participant 1). However, there are other variables that matter as well. For instance, participants recognized that they had to weigh the cultural and community values when selecting a program to implement. For instance, one participant explained, “That’s been a challenge… A lot of the PBIS research is based on Anglo, you know, American, but you have to mix in that, that cultural piece” (Participant 10). Additionally, participants highlighted the importance of having factors that increase the potential social validity of a program. One participant highlighted that research-based programs help secure stakeholder buy-in:

“We try to stick to interventions … that are research based, because we can tie it back with …. you know…stakeholders.”

(Participant 9)

The idea of “stakeholders” also included factoring in the parental perspectives and values of the local context when selecting a mental and behavioral health support program. For instance, many participants suggested that this serves as a critical filtering factor, as successful implementation depends on alignment with the expectations and concerns of families. In a similar sense, the study found that the strength of relationships between district and school staff impacted the implementation of programs. For instance, when relationships between staff were strained or lacked alignment, teacher buy-in decreased and implementation fidelity was compromised. One participant explained:

“So…if we, have a teacher in the building who doesn’t believe in the program, …which then impacts decision making.”

(Participant 12)

Another key driver of the filtering process was the local, state, and federal mandates related to behavioral intervention programs and the associated compliance. Navigating mandates has continually impeded implementation efforts but has been amplified in recent years due to political factors (McCarthy et al., 2026). Participants discussed that legal constraints around the conversation of mental and behavioral health often restricted the decision-making process. For instance, one participant stated, “I think it [the decision-making process] goes umbrella down, and so we have state policies, I mean… that we can only talk about certain things, ask certain things, you know?” (Participant 15). Relatedly, participants had to measure potential programs by their compatibility with existing school procedures and internal compliance expectations based on mandates. In many instances, programs that fit within the current operational structure of the school are prioritized by the school district. One participant provided a descriptive explanation of filtering through local and state laws to ensure that a program can fit within their context:

“What are our policies that we have in place and am I following it? …what’s our accountability if we do this? Who’s going to sue us or not give us funding?”

(Participant 12)

Finally, the idea of “resources” continually came up as a major factor in determining if a school can implement a program. Specifically, funding and time were mentioned as resources that directly impact the adoption and implementation of a program. Many participants remarked that funding for appropriate levels of trained staff has been a long-term barrier for school districts; however, some noted that there are “hidden fees” related to implementation that are often not considered:

“So sometimes they’ll be like, Oh, it’s a totally free thing. But then as you’re going through the activities, they’re like, yeah, and you’re going to need 50 pipe cleaners…it’s usually not just a one and done…So also looking at, like long term, what is the actual cost going to look like?”

(Participant 2)

Relatedly, time was often cited by participants as the most constrained resource, shaping a program’s implementation. Additionally, there was a general trend of behavioral and mental health programming being deprioritized within schools. For reference, one participant stated, “There’s only so much time and all the demands of academics.” (Participant 3) and another reflected on the unfortunate reality of prioritizing student time.

“…when you think about adding…instructional minutes to directly teaching SEL [social-emotional learning]…it is one of the first things on a busy day to not be taught.”

(Participant 13)

The final “resource” consistently discussed was staff availability and capacity. Regarding staff availability, participants expressed that limited availability of staff impacted the scope and fidelity of intervention implementation. Thus, school decision-makers must look at staff roles and their capacity to implement a program. For example, one participant who was an administrator described continually monitoring the capacity of staff when determining which intervention to implement: “I’m constantly looking at the capacity of my staff to identify” (Participant 10). Beyond the availability of staff, access to professional development can support effective adoption and implementation of a program within the school setting.

In sum, the filter substage is a multi-faceted component of this model that requires school decision-makers to consider a variety of factors prior to the final substage of “Select”. The duration and depth of this substage are contextually dependent, but once it is completed, school decision-makers move on to the second stage of the model, Trial.

#### 3.2.2. Stage #2: Trial

The Trial Stage of the STAR model focuses on a small and targeted implementation of the selected program from the “Select” stage of the model. Specifically, once a program has successfully been filtered through the noted variables in the last section, the program is then selected to be implemented within its localized setting. Many participants referred to this part of the model as the “pilot” of a program that is selected as described below:

“…trialing it informally with like one student or two students, and then as a team, see success with the one or two students…getting a few schools buy ins, and then being able to present our local data of how effective this has been. Then district wide, when we roll, roll it out bigger.”

(Participant 14)

Within the implementation science literature, piloting or trialing of a program or new practice is a critical step for effective adoption within a local context (Fixsen et al., 2010). The piloting of a program was often described as pre-determined schools or individuals using the new program within their setting to see if the program is feasible and has social validity. However, this process was unsystematic and informal as one participant described “… a lot of trial by fire” (Participant 5).

Within this stage, there are three sub-themes that reflect key components of what the trialing of a program would entail within the respective school settings. First, the notion of “de-implementation” (i.e., replacing a current practice with a new practice) was necessary when school teams attempt a new practice. Given that educators are often accustomed to never-ending changes that result from new initiatives (i.e., “change fatigue”; Koh et al., 2023), a general theme was building on old practices, as opposed to adding to old practices. One participant provided an example of how PBIS has changed throughout the years:

“PBIS came around about 13–14, years ago in our district…everybody came to the same workshop and walked away with posters and rewards…So this time, we’ve really reinforced that, hey, we’re we’re talking about posters and tickets…but…it’s that responding the behavior and using consequences to change behavior… We know that this burned you before we’re we’re not saying you’re not going to get burned again, but our goal is to do this piece…”

(Participant 13)

Second, school decision-makers expressed that a new practice often needs a “champion” to be the resident expert and marketer of the program to increase the adoption of the new practice. This person was not in a particular role or position in a school, but was identified given their charisma, strong relationships with other staff and effective communication skills. One participant highlighted, “looking for those champions, who’s going to carry that banner….” (Participant 9).

Finally, mirroring the themes from the first stage participants discussed the need for ongoing professional development and coaching to increase the capacity of school staff to not only trial the new program but also assess its potential for sustainability.

#### 3.2.3. Stage #3: Assess

Upon completion of trialing the program (Stage #2), schools then move to the third stage of the STAR model which is Assess. This stage focuses on (a) how staff perceive the selected program and (b) the localized evidence of the program. First, rather than focusing primarily on implementation fidelity, participants emphasized whether the program appeared effective within their specific school context depended on how useful and manageable the program appeared. As one participant explained, staff often focus on the input (i.e., time needed) and the output (i.e., effects of a program) when it comes to assessing the worth of a program.

“barriers are always it’s just one more thing, right? We’re putting one more thing on somebody’s plate, though…how do we help the perception to be this is this is going to be important?”

(Participant 12)

Second, the localized evidence of the program was determined based on how the program impacted indicators of student achievement. A main variable that swayed the perceived localized evidence of a program was data that was presented within team meetings or school-wide meetings. One participant even suggested that buy-in increased when teachers personally observed meaningful changes associated with the innovation, “…the adult educators, start to see the value in the data. They start to…believe in the practice to see the change.” (Participant 13).

Both themes were noted within observations. First, teams had conversations about how a specific intervention is being received by staff. However, there often was an absence of implementation data such as fidelity and student outcomes data with a heavy reliance on staff providing a narrative reflection of how a program was being implemented.

#### 3.2.4. Stage #4: Revise

Finally, the fourth stage of the STAR model is Revise. This is where school decision-makers decide if they need to review (i.e., dive deeper), revise (i.e., change the program), or repeat (i.e., increase implementation) the selected program. Ongoing refinement of a new practice is both a practical expectation and is outlined within the implementation science literature (Kilbourne et al., 2024; Ryan et al., 2024). The first decision of review is instances where a program showcased some success; however, further investigation of this program is necessary for sustained implementation. For instance, one participant outlined how their district implements check in and check out but needed to modify it to better support their students.

“we do a lot of check in and check out…but we’re finding even check and check out is not enough…We also have really shifted towards skill streaming…Breaks are Better.”

(Participant 9)

Within the observations, there were multiple instances of staff members wanting to modify the intervention to make it more effective within their setting. Specifically, noting that the intervention program would work if there is more localized connection to the implementation procedures.

Regarding the decision for revising a program, schools would focus on changing the program as a whole and potentially begin again at the first stage of the STAR Model. This decision would be driven by both poor perceptions of the program and a lack of local evidence of the program. One participant explained how revising implementation often involved anticipating future needs and adjusting supports accordingly, stating, “Even for next year, like we’re going to roll off this new program, it’s going to cover more information that we haven’t been providing in the past.” (Participant 1).

Finally, the choice of repeating a program would be instances where a school finds success in both the perceptions of the program and the local evidence of the program. This would encompass a more formal and large-scale rollout of the program across different settings such as implementation in more classrooms and/or schools. Overall, regardless of the choice, participants noted that this stage is often creative (e.g., staff needing to utilize limited resources), flexible (e.g., success is variable), and iterative (e.g., assumption of refinement).

## 4. Discussion

School systems may be the most accessible and effective avenue for delivering evidence-based mental and behavioral health services to youth (Sanchez et al., 2018). However, implementation is constrained by challenges related to staff capacity, competing priorities, and the translation of research evidence into routine practice (Lyon et al., 2024). The findings from this study offer an interpretive account of how a group of school decision-makers described selecting and implementing mental and behavioral health supports within their local contexts. From these accounts, the STAR model was developed as a contextually grounded representation of participants’ decision-making processes. Rather than proposing a universally applicable implementation framework, the model represents an initial contextualization that may inform future research examining how school teams navigate complex implementation decisions across varied educational settings.

### 4.1. Findings

The decisional processes described were shaped by specific structural conditions that limited the available choices before team deliberation. For example, participants described state policy that limited what could be discussed (Participant 15). They described liability and funding contingencies that functioned as hard constraints, asking “Who’s going to sue us or not give us funding?” (Participant 12). These pressures and constraints were situated within a broader socio-political environment in which mental and behavioral health services have become increasingly questioned (McCarthy et al., 2026). These conditions are essential parts of the STAR model and illustrative of school decision-making within a bounded rationality framework. Policy and decisional power operate upstream of the filtering substage by determining which programs are even allowable, while school level constraints (e.g., competing mandates, capacity) dictate which of the programs are feasible to implement. These factors determine the parameters within which the STAR process unfolds. Implementation supports intended to improve school decision-making must account for these conditions that often are outside the control of school decision-makers.

### 4.2. Implications

Throughout the development of the STAR model, there were multiple implications that may connect to the current implementation gaps within school-based systems of support (i.e., MTSS). First, many schools intend to implement effective processes for the implementation of mental and behavioral health programs within their school systems. Specifically, they are intentional in their design and critical in evaluating the effectiveness of their choices and processes. This was evident through the filtering substage within the model, in which school decision-makers filter a decision through multiple factors (i.e., community values, policies, and current resources) prior to deciding to implement. With that said, research also suggests that to achieve the intended outputs of an MTSS framework (e.g., improved student mental and behavioral outcomes), one must ensure high-quality inputs (e.g., evidence-based programs aligned with a school’s needs and capacity). Therefore, participants’ experiences suggest that school teams may benefit from support that helps achieve MTSS-related goals, while connecting it to the local context.

Second, the experiences of participants suggest that educators are committed to implementing programs that will support all students within their MTSS framework. This is evident within the Trial and Assess stages of the model, in which educators examine the localized results (i.e., how the program worked in the local context) and staff perceptions (i.e., how teachers and staff perceived the program) as related to the problems that are most prevalent within the setting. Research also indicates that several factors can influence MTSS effectiveness, including how educators use data to inform their efforts (C. N. Taylor et al., 2018) and the fidelity with which MTSS-based practices are implemented (Flannery et al., 2014; McDaniel et al., 2022). However, one could argue that the design of an MTSS framework is most fundamental to its potential success. For instance, an MTSS framework is unlikely to be sustainable or yield intended outcomes if it includes low-value practices or impractical strategies (Farmer et al., 2021). Third, and related to the previous point, the findings suggest that educators want access to information that will inform their MTSS design, which is directly related to the information gathering subsection. Often, reputable information is available, but it is not always accessible, such as when it is behind journal paywalls or presented at academic conferences. Fourth, even when high-quality information is available, it is not always clear to educators how to apply it to their context. Within the STAR model, the final stage (i.e., Revise) is often creative, flexible, and iterative due to the uniqueness of each environment.

Fifth, participants in the present investigation depended heavily on local evidence and direct observation of change to evaluate the effectiveness of programs and services. This reliance is consistent with predictions from a bounded rationality framework and carries generalizability risks, despite the potential for improved contextual fit. For example, local evaluation of evidence may be subject to confirmation bias, infer effectiveness from small and non-representative samples, and mistake regression to the mean as an indicator (or not) of actual program effect, each of which may result in the misuse of limited resources to continue or scale programming. It is rarely the case that decisions are made in the true absence of “evidence,” rather than that reliance on weak or indefensible evidence sources may undermine intended outcomes. The STAR model surfaces these expected constraints to inform how decision-makers may identify and acknowledge these processes to better account for potential impacts on outcomes.

Finally, the STAR model provides direct comparisons to existing implementation logic and frameworks, while providing a more nuanced viewpoint. For instance, related to the Fixsen et al. (2024) implementation model, there were major similarities such as both models having (a) a stage of exploration (i.e., Select vs. Exploration), (b) a stage of installing (i.e., filtering vs. Installation), (c) a stage of initial implementation (i.e., Trial vs. Initial Implementation) and multiple implementation drivers (i.e., coaching, professional development, alignment). In the same sense, the STAR model also closely mirrors the EPIS model (Exploration, Preparation, Implementation, and Sustainment; Aarons et al., 2011). The main areas in which the STAR model deviates from these established models are (a) the reality of localized resources and (b) the specific focus on mental and behavioral health implementation. The STAR model was developed with a bounded rationality model that recognizes that school-based decisions are unlikely to be optimized for a particular outcome as it is not possible to critically evaluate all options. Thus, this framework attempts to add to existing models to provide a more contextualized model for decision-makers to navigate implementation. Finally, the STAR model focuses on the unique context of implementing mental and behavioral health supports within schools. The implementation science framework and EPIS model are more broadly defined, which can be applicable; however, the STAR model provides a more updated and nuanced viewpoint of behavioral health implementation in schools.

### 4.3. Limitations

There are several limitations of the present study. First, the relative thinness of observation data in comparison to interview data limited more nuanced comparisons. The study was designed to collect rich descriptive observations of decision-making in natural contexts, but not all observers included descriptive memos and instead relied on check boxes to report whether something was observed (e.g., data-based decision-making). As such, the STAR model may not fully reflect the contexts and conversations in which decisions are made in real time and restricts the ability to examine the enactment of participants’ insights. Additionally, the reliance on self-reported interviews may have led to retrospective rationalization, professional ideals, and/or socially desirable descriptions rather than actual decision practices. Additionally, the lack of observational data from Pennsylvania may have affected the development of the model, since the researchers did not observe real-life decision-making and only based the model on the personal reflections of participants. Overall, the imbalance in data sources and reliance on self-report may also shape how decision-making processes are represented within the model, particularly given the interpretive and process-oriented nature of the analytic approach. Future observations of decision-making meetings are planned in future academic years to support further model refinement. The interviews did yield thick descriptions of people, places, and events of mental health decision-making, which lends initial contextual credibility to the STAR model. However, future studies will include a more balanced approach of multiple qualitative data sources and opportunities to compare the conceptualization and enactment of participants’ perspectives.

Second, observations were conducted exclusively in elementary and middle schools. The STAR model may function differently in high schools with varying forms of resources, capacity, mandates, as well as staff relationships. Participants from the districts across three states reflected varying levels of MTSS implementation, and those schools with less established MTSS may engage in only part of the STAR model. In the same sense, the sample for the study was profession-specific and purposefully sampled, with all participants in an administration or student support role. Thus, the STAR model may reflect an idealistic and narrowed viewpoint due to missing perspectives of teachers, families, students, and district-level policymakers. The inclusion of these stakeholders might increase the applicability of the model to daily decision-making. While the interviews included participants across rural, suburban, and urban districts, resource availability varied widely. The STAR model may offer processes that are transferable but substance that is context-dependent. This status is consistent with provisional process models and transferable guidance from qualitative research (Lincoln & Guba, 1985). Future studies should include more stakeholders (i.e., teachers, families, students, and district-level policymakers) and more diverse settings to make the model more universally applicable.

Third, future research will be necessary to engage in member-checking (Lincoln & Guba, 1985) and outside expert evaluation of the STAR model with new decision-makers and school contexts. Therefore, the STAR model as presented is considered provisional, available for revision based on feedback from those who theorize, study, and make mental-health programming decisions in K-12 schools. Additionally, as the model was developed through an iterative and interpretive analytic process, it reflects the research team’s co-constructed understandings of the data, which may limit reproducibility across researchers or contexts. Further, the recursive and exploratory nature of this analytic process may make it more difficult to isolate discrete analytic steps, with implications for transparency and consistency. As such, the findings should be interpreted as a contextually grounded and empirically informed model that remains open to refinement as it is examined across additional settings and perspectives. These limitations point to important next steps, including engaging in member checking and expert review, as well as examining the model across diverse school contexts to further refine and broaden its applicability.

### 4.4. Recommendation for Future Action

In the development of the STAR model, it also became apparent that educators could benefit from tools that (a) vet and curate high-quality evidence, and (b) present this information in a manner that is consumable and can be used to easily inform MTSS design efforts. For instance, participants may benefit from tools to help them determine whether it is appropriate to adopt an intervention given the school’s available staff and resources. This would be most applicable within the “Select” stage of the model. Those same tools could provide information on an intervention’s anticipated outcomes, as well as the logistics of its implementation (e.g., cost, number of required staff, time required to implement each day or week, anticipated duration of implementation) to better inform the “Trial” or “Revise” stages. This would result in allowing a school to move from simply knowing of an intervention program to knowing the potential of an intervention program to work as related to their local context. Thus, the STAR model can provide a guiding basis of knowledge, paired with tools for schools to navigate the different stages of implementation and decision-making.

## 5. Conclusions

The findings of this study provide additional avenues for further research and examination of decision-making for mental and behavioral health services within schools. The STAR model is a novel and preliminary result of this study and should be further applied in school-based decision-making to guide refinement and increase applicability. For instance, this study is just the initial development of the STAR model. Future studies could further examine its applicability with different decisions within schools, as well as the perspectives of different stakeholders and experts to support its use as a guiding framework for decision-making in schools. Additionally, the STAR model further amplifies the need for tools that help (a) translate research evidence, (b) model the impact, cost and implementation of decisions, and (c) provide more consumable and understandable information on the research evidence of mental and behavioral health programs. As the bounded rationality of school decision-makers will continually vary, innovative tools and connected information can act as a broker of information to better close the gap between research evidence and the local context for implementation.

## Data Availability

The data presented in this study are not publicly available due to privacy and ethical restrictions protecting participant confidentiality. Access to the data is restricted to protect the anonymity of participants involved in the study. Data may be made available by the corresponding author upon reasonable request, subject to institutional and IRB approval.

## Supplementary Materials

The following supporting information can be downloaded at: https://www.mdpi.com/article/10.3390/bs16081319/s1, Figure S1: STAR Model of Behavioral Health Implementation in Schools; Figure S2: Diagramming Example; Figure S3: Digital Diagram Example; Figure S4: Interim Omnibus diagram; File S1: Interview Protocol; File S2: Observation Protocol; Table S1: Quantity of Theme Instances; Table S2: Summary of the STAR Model of Behavioral Health Implementation in Schools.

## Abbreviations

The following abbreviations are used in this manuscript:

STAR	Select, Trial, Assess, Revise
MTSS	Multi-Tiered Systems of Support
PBIS	Positive Behavioral Interventions and Supports
IRB	Institutional Review Board
IDA	Iterative Diagrammatical Analysis
BSEL	Behavioral and Social Emotional Learning
IEP	Individual Education Plan
