# Supplementary material for: From Choose and Hope to Evidence and Impact: Rethinking School-Based Behavioral Health Decisions
Source: Behav Sci (Basel). 2026 Aug 3;16(8):1319. doi: 10.3390/bs16081319 (PMC13509306; doi:10.3390/bs16081319)
Supplement: Supplementary file 1 [file behavsci-16-01319-s001.zip › File S1.pdf]

Introduction (*5 Minutes*)

Once the participants enters the virtual meeting:

- Ensure they have read the consent form and sign
  - *"Have you had a chance to review the consent form? Do you have any questions or concerns with what you are participating in?"*
- Introduce the interview team
  - *"For this interview, we will have a facilitator (myself) and a note taker, which is [Insert team member's name]."*
- Outline the Purpose and Process of the Interview
  - *"The purpose of this project is to understand how school-decision makers use research to inform decision-making specific to mental health interventions/assessments. We will be asking about your experiences and gather your reactions specific to mental health interventions/assessments."*
  - *"This interview will take approximately 60 minutes. Everything discussed today will be kept confidential. Your specific responses will not be shared with anyone outside of our team. We are video-recording and transcribing this session only as a tool to capture all information. Any questions?"*
    - Answer any questions provided by the participant
  - *"Alright, we are going to turn on the recording and transcription software and begin the interview"*
    - Turn on recording and transcription software

Demographic and School Context (approx. *20 minutes*)

*Solicit feedback via the probes below

- Tell us about your role and the school that you work in.
  - *How long have you been in your current role at your school? Before this school? (Prompt for education, training, and experience)*
  - *Please describe your school. What makes your school special or unique?*
  - *What are the main responsibilities of your role?*
- Who would you say are the primary decision-makers at your school?
  - *What is it like being a decision-maker at your school?*
  - *How long have you been in your current role at your school? Before this school? (Prompt for education, training, and experience)*
- What mental health issues do you see as most pressing at your school?
  - *What makes these issues so pressing?*
  - *What do you believe should be done at school to support these issues?*
- How are students identified for needing mental health support?
  - *To what extent do you find these methods effective?*
- Please discuss any mental health programs/curricula/interventions at your school.
  - *What do you think about them?*
  - *Does staff value these programs?*
  - *How did you decide which programs/curricula/interventions to use?*

<Share screen with summary document (See Last Page) and place document in the chat>

"I just placed a document within the chat that I will share on my screen. This document outlines variables that typically impact decision-making for mental health programs within schools."

- Please take a minute and **select three** that you find to have the most impact on **decision-making for mental health programs** at your school.
    - *Which three did you choose? Please provide rationale.*
    - *How do these variables impact decision-making for mental health programs at your school?*
    - *Which one of your top three is the most impactful and why?*

"Any other comments regarding mental health programs within your school?"
<Interview facilitator summaries main points, ask for clarification (if necessary)>
"*Now we are moving on to discuss the use of research evidence within your school"*

Values and Beliefs about Research Evidence (approx. *20 minutes*)

- What do you define as "research-based"? What does "research evidence" mean to you?
    - *How do you access research evidence? How do you know something is 'evidence-based?'*
        - *If there is a reference to a specific document>*
            - *Can you provide more information on <insert document name>?*
            - *Would you mind sharing that document with us after the interview?*
    - How accessible are "research-based" resources to inform your decision-making?
        - *Where would you go to find "research-based" resources?*
        - *Who at your school provides "research-based" resources?*
- How important is research evidence in your decision-making for school-based mental health services?
    - *How do use research evidence in your decision-making for school-based mental health services*

<Return back to the variable summary document>

"I am going to jump back to the document I shared with you previously."

- Please take a minute and **select three** that you find to have the most impact on **accessing research evidence for mental health programs** at your school.
    - *Which three did you choose? Please provide rationale.*
    - *How do these variables impact accessing research evidence for mental health programs at your school?*
    - *Which one of your top three is the most impactful and why?*

"Any other comments regarding research evidence within your school?"

<Interview facilitator summaries main points, ask for clarification (if necessary)>

"*Now we are moving on to discuss the decision-making process"*

Decision-Making Process (approx. *20 minutes*)

"Take a second and think about a recent mental health program/curricula/intervention decision you made or are making at your school"

- Please explain the mental health program/curricula/intervention decision you were just thinking about.
  - o *Please describe the people involved in the decision-making process.*
  - o *How do you engage with other staff (i.e., leadership team) and educators (i.e., teachers) at your school regarding the decision-making process?*
  - o *Please note the timeframe of the program, is it currently being implemented?*
- Can you please share your decision-making process? What did you do first, second, third, and so on?
  - o *What factors were most important to consider?*
  - o *What barriers did you face?*
- What research evidence-informed your decision?
  - o *What 'artifacts' (documents, presentations, videos, websites, etc…) can you share that informed your decision-making?*
- How do you feel about your decision-making process?
  - o *What was most difficult?*
  - o *What was easiest?*
  - o *What advice would you give to a new decision-maker trying to decide on a mental health intervention for their school?*
- How does funding or allocation of funds impact your decision-making process?
  - o *How was the allocation of funds determined? Please provide rationale.*
  - o *What was prioritized for the allocation of funds? Please provide rationale.*

"You mentioned a few barriers to the decision-making process you outlined. We would like to get your perspective on a common barrier faced by school-based decision-makers. Specifically, instances where resources and staff capacity are constrained, resulting in a limited ability to carry out implementation. For instance, the number of students that demonstrate the need for a mental health program exceeds a staff's capacity to implement a mental health program"

- Have you experienced this barrier in your school?
  - o *How do you address this?*
  - o *What would you consider as a solution to this problem?*
  - o *How does this impact the decision making process?*

"Any other comments regarding research evidence within your school?"
<Interview facilitator summaries main points, ask for clarification (if necessary)>
"*Now we are moving on to some observational data we collected"*

Conclusion (*5 minutes*)

<Interview facilitator summaries main points of all the interview,>

"Is that correct? Is there anything else anyone would like to add"

"Thank you so much for sharing your thoughts with us! If other ideas occur to you after this meeting, please contact us at the number provided on your consent form. (or give alternative contact info)."

<u>Variables that Impact Decision Making</u>

Social Negotiation	*Example: Relationships with Other Staff*
Personal Beliefs and Values	*Example: Belief of Program Impact*
School Resources	*Example: Staff Capacity and Resources*
Political/Societal Factors	*Example: Concerns Addressed By The School Board and/or Community*
Community Factors	*Example: Home-School Connection*
District Level Policies	*Example: Polices for School-based Mental Health*
State Level Policies	*Example: Accountability Mandates*
