# Supplementary material for: From Choose and Hope to Evidence and Impact: Rethinking School-Based Behavioral Health Decisions
Source: Behav Sci (Basel). 2026 Aug 3;16(8):1319. doi: 10.3390/bs16081319 (PMC13509306; doi:10.3390/bs16081319)
Supplement: Supplementary file 1 [file behavsci-16-01319-s001.zip › File S2.pdf]

Document A: Tiers I & II Observation Checklist		
Date: 3/27/25	***Start Time of Observation:***	***End Time of Observation:***
Observer:		
School:		

Directions: At the beginning of the observation (i.e., 3-5 minutes into the meeting), indicate whether each of the personnel identified in items 1-9 were present or absent. For items 10-20, please indicate whether the critical component of problem-solving was present or absent during the observation. These items can be completed during or immediately after the observation. For items 10-20, please input a general description of either the presence or absence of each item.

Critical Component	Present?	Absent?	Evidence/Notes
Personnel Present			
1. Administrator			
2. Classroom Teacher			
3. Parent			
4. Data Coach			
5. Instructional Support (e.g., Reading Coach)			
6. Special Education Teacher			
7. Facilitator			
8. Recorder (i.e., Notetaker)			
9. Timekeeper			

Critical Component	Present?	Absent?	Evidence/Notes
Problem Identification			
10. Data were used to determine the effectiveness of Tier I instruction			
11. Decisions were made to modify Tier I instruction and/or to develop supplemental (Tier II) interventions			
12. Universal screening (e.g., DIBELS, ODRs) or other data sources (e.g., district-wide assessments) were used to identify groups of students in need of supplemental intervention			
13. The school-based team generated hypotheses to identify potential reasons for students not meeting benchmarks			
14. Data were used to determine viable or active hypotheses for why students were not attaining benchmarks			

Critical Component	Present?	Absent?	N/A	Evidence/Notes
Intervention Development/Support				
15. Modifications were made to Tier I instruction<br>***(Note: Select N/A under the Evidence/Notes column for a-c if a defensible decision was made to NOT modify Tier I instruction)***				
a. A plan for implementation of modifications to Tier I instruction was documented				
b. Support for implementation of modifications to Tier I instruction was documented				
c. Documentation of implementation of modifications to Tier I instruction was provided				
16. Supplemental (Tier II) instruction was developed or modified<br>***(Note: Select N/A under the Evidence/Notes column for a-c if a defensible decision was made to NOT modify Tier I instruction)***				
a. Plan for implementation of supplemental instruction was documented				
b. Support for implementation of supplemental instruction was documented				
c. Documentation of implementation of supplemental instruction was provided				

Critical Component	Present?	Absent?	Evidence/Notes
Program Evaluation/RtI			
17. Criteria for positive response to intervention were defined for school-, grade- or classroom-wide interventions			
18. Progress monitoring and/or universal screening data were collected/scheduled			
19. A decision regarding School-, grade- or classroom- wide RtI was documented			
20. A plan for continuing, modifying, or terminating the intervention plan was provided			

Additional Comments:

Document B: ADVICE Project Note Catcher		
Date:	***Start Time of Observation:***	***End Time of Observation***
Observer:		
School:		

Setting
Please note (a) the location of the meeting, (b) the layout of the room, and (c) your general perspectives of the meeting functioning.

Persons In Attendance
Please note who is at the meeting (using professional titles only), if certain positions are absent, and the perceived roles of the group members (e.g., who is leading the meeting).

Physical Actions And Non-Verbal Gestures
Please note your perspectives on the non-verbal communication between and across team members. Examples may include body language, non-verbal reactions to meeting discussions, and/or team members' demeanor.

Discourse
Please detail the discussion of the meeting and your perspective(s) of the meeting's discussion. Examples may include your perspectives on the functioning of the meeting, a detailed explanation of the conversation between and across team members, and emotions tied to the discussion. Refrain from documenting conversations of specific student-level data.

Multi-Tiered Systems of Support for Student Mental Health
Please note any reference or discussion to Multi-Tiered Systems of Support (MTSS) for Student Mental Health. Examples may include discussion around the implementation of MTSS, Tier I and II supports provided, data to determine the effectiveness of Tier I and II services, and interventions or assessments encompassing the MTSS decision-making process.

Mental Health Interventions
Please note any reference or discussion to intervention program(s) intended to address mental health concerns (i.e., behavioral and/or emotional). Please document the intervention, the conversation about the intervention, and general impressions of the team's knowledge of the intervention. Examples may include discussion around the implementation and/or planning of an intervention program, classroom and/or school-wide data collection, and/or progress monitoring.

Mental Health Assessments
Please note any reference or discussion to an assessment or assessment data intended to address mental health concerns (i.e., behavioral and/or emotional). Please document the assessment, the conversation about the assessment, and general impressions of the team's knowledge of the assessment. Examples may include discussion around the implementation and/or planning of an assessment administration, classroom and/or school-wide data collection, and/or review of the assessment data.

Decision-Making
Please note any reference or discussion regarding the process of decision-making for implementing intervention program(s) and/or assessment(s) intended to address mental health concerns (i.e., behavioral and/or

emotional). Some examples may include your perspectives on how the team came to a decision for Tier I and II supports, discussion of data to make a decision, and/or logistical considerations for decision making (i.e., time and resources).

Use of Research Evidence

Please note any reference or discussion using research or research-based practices related to assessment(s) or intervention program(s) intended to address mental health concerns (i.e., behavioral and/or emotional). Some examples may include reviewing published articles or research briefs, discussing research-based requirements for intervention or assessment, and/or incorporating research or research-based practices into typical school practices for addressing mental health concerns.

General Impressions

Please reflect on the functioning of the meeting and provide comments on your overall perspective of the team's use of research evidence and decision-making for intervention program(s)and/or assessment(s) intended to address mental health concerns. Some examples may include your perspective of the team's strengths, topics not discussed that you find pertain to the meeting's purpose, and/or unclear situations or limitations to your observation.
