# Supplementary material for: From Choose and Hope to Evidence and Impact: Rethinking School-Based Behavioral Health Decisions
Source: Behav Sci (Basel). 2026 Aug 3;16(8):1319. doi: 10.3390/bs16081319 (PMC13509306; doi:10.3390/bs16081319)
Supplement: Supplementary file 1 [file behavsci-16-01319-s001.zip › Table S1.pdf]

**Table 1.**

*Quantity of Theme Instances*

| Theme | | Number of Participants Mentioning Theme | Example Quote of Theme |
|---|---|---|---|
| Core Elements | Empowerment of Teachers/Implementers | 9 | "Teachers to make the decision and have the knowledge base to make decisions to support their students because they know them best" (Participant 10). |
| | Alignment | 7 | "We're trying to align that with whatever the BSEL (Behavioral and Social Emotional Learning) goals are on the IEP" (Participant 12). |
| | Relationships | 15 | "…if we develop those relationships with the families, it makes some of our decision making, I think, a little bit easier…" (Participant 6). |
| | Trust | 16 | "Is it from someone in education, a PhD? Is it in an academic journal? What are, what's the data behind it?" (Participant 5). |
| Need for Change | Alignment with Mission and Vision of the School/District | 8 | "…determine what kind of a plan would best fit us as a district and move forward from there" (Participant 5). |
| | Noted Problem or Specific Need | 14 | "If attendance is an issue, you're going to end up having some concerns, whether it's because of mental health or because of something else…" (Participant 1) |
| | Policy and Mandates | 12 | "…can they (i.e., components of a selected program) meet these requirements…?" (Participant 4). |
| | Innovative or Promising Practice | 4 | "We've had so many initiatives, and I think about it all the time. And those shiny new things are great, but oftentimes we just don't have the again, back to capacity. We just don't have the capacity to sustain it…"(Participant 9). |
| Stage #1: Select | Information Gathering | 16 | "I'm probably gonna do a Google Scholar search on research that's been done on that, on interventions that have been done with that, and then kind of pull some and then probably analyze |

| | | |
|---|---|---|
| | | the what's most similar in all of that research to help determine what strategies are the most similar." (Participant 12). |
| Filter | 11 | "That's been a challenge... A lot of the PBIS research is based on Anglo, you know, American, but you have to mix in that, that cultural piece" (Participant 10). |
| Stage #2: Trial | 9 | "…trialing it informally with like one student or two students, and then as a team, see success with the one or two students, training them more on how to do it more independently, getting a few schools buy ins, and then being able to present our local data of how effective this has been. Then district wide, when we roll, roll it out bigger." (Participant 14) |
| Stage #3: Assess | 9 | "...the adult educators, start to see the value in the data. They start to...believe in the practice to see the change. And then my buildings that have seen that, not just at the leadership level, but in the classrooms, have really bought … those tier two practices." (Participant 13) |
| Stage #4: Revise | 10 | "We do a lot of check in and check out, which is great, but we're finding even check and check out is not enough…We also have really shifted towards skill streaming…Breaks are Better where it's check in, check out, and we're adding in the breaks for kids, because we're finding check in, check out is not enough." (Participant 9) |
