# Supplementary material for: From Choose and Hope to Evidence and Impact: Rethinking School-Based Behavioral Health Decisions
Source: Behav Sci (Basel). 2026 Aug 3;16(8):1319. doi: 10.3390/bs16081319 (PMC13509306; doi:10.3390/bs16081319)
Supplement: Supplementary file 1 [file behavsci-16-01319-s001.zip › Table S2.pdf]

Table 2

Summary of the STAR Model of Behavioral Health Implementation in Schools

Model Component	Definition	Participant Quotes
**Foundational Component 1:** Core Elements	Core elements are contextual conditions that support the adoption and transition of behavioral health practices into routine school practice.	
Empowerment	Teachers and implementers are empowered to make informed decisions, assume leadership, and take ownership of implementation.	"Teachers...have the knowledge base to make decisions to support their students because they know them best." (Participant 10)
Alignment	Interventions are evaluated based on their fit with existing school goals, structures, initiatives, and student support systems.	"They try...to link it to our school wide system." (Participant 10)
Relationships	Strong relationships among educators, specialists, administrators, students, families, and community partners facilitate communication, collaboration, and implementation fidelity.	"…if we develop those relationships with the families, it makes some of our decision making, I think, a little bit easier…" (Participant 6).
Trust	Trust in colleagues, leadership, and the evidence supporting an intervention influences implementation decisions.	"...what's the data behind it?" (Participant 5)
**Foundational Component #2: Need for Change**	A recognized need or catalyst initiates consideration of a new behavioral health practice or intervention.	
Mission and Vision	New practices are evaluated based on consistency with the school's or district's mission, vision, and strategic goals.	"...determine what kind of a plan would best fit us as a district." (Participant 5)
Identified Need	Student needs, behavioral concerns, attendance patterns, or other local data create urgency for implementation.	"If attendance is an issue, you're going to end up having some concerns, whether it's because of mental health or because of something else…" (Participant 1)
Policy and Mandates	Federal, state, and district policies shape which interventions can be selected and implemented.	"...can they meet these requirements?" (Participant 4)
Innovation or Promising Practice	New or emerging practices stimulate interest but must be balanced against local capacity and sustainability.	"Those shiny new things are great, but...we just don't have the capacity to sustain it." (Participant 9)
**Stage 1: Select**	School teams gather information, evaluate evidence, filter options through local conditions, and select an intervention for implementation.	"I'm probably gonna do a Google Scholar search...to help determine what strategies are the most similar." (Participant 12)

**Stage 2: Trial**	Selected interventions are piloted to determine feasibility, contextual fit, and social validity before broader implementation.	"...trialing it informally with like one student or two students...then...roll it out bigger." (Participant 14)
**Stage 3: Assess**	School teams assess both staff perceptions and localized evidence to determine whether the intervention is meeting intended outcomes.	"...educators start to see the value in the data...believe in the practice to see the change." (Participant 13)
**Stage 4: Revise**	Based on assessment findings, interventions are refined, expanded, or replaced through an ongoing, iterative process.	"...check in, check out is not enough...we've shifted toward skill streaming." (Participant 9)
